# Enhanced Therapeutic Effects of an Antitumor Agent on Subcutaneous Tumors in Mice by Photomechanical Wave-based Transvascular Drug Delivery

**DOI:** 10.7150/jca.84066

**Published:** 2023-06-19

**Authors:** Yasuyuki Tsunoi, Hitoshi Tsuda, Satoko Kawauchi, Koji Araki, Shunichi Sato

**Affiliations:** 1 Division of Bioinformation and Therapeutic Systems, National Defense Medical College Research Institute, Japan.; 2 Department of Basic Pathology, National Defense Medical College, Japan.; 3 Department of Otolaryngology-Head and Neck Surgery, National Defense Medical College, Japan.

**Keywords:** photomechanical wave, laser-induced stress wave, transvascular drug delivery, drug delivery system, cisplatin, chemotherapy

## Abstract

**Purpose:** We previously developed a site-selective transvascular drug delivery system based on nanosecond pulsed laser-induced photomechanical waves (PMWs). In this study, we applied this method to the delivery of cisplatin (cis-diamminedichloroplatinum, CDDP) to a subcutaneous tumor in a mouse and examined its antitumor effects.

**Methods:** A mouse tumor model with subcutaneous inoculation of human head and neck cancer cells (FaDu cells) was used. The mice were divided into four groups: control without any treatment (control), CDDP application only (CDDP only), PMW application only (PMW only) and combined application of PMWs and CDDP (PMW+CDDP). A PMW was generated by irradiating a laser target, which was placed on the skin over the tumor, with a ruby laser pulse (fluence, 1.6 J/cm^2^). A CDDP solution was intraperitoneally injected into the mice (2.5 mg/kg).

**Results:** Until 7 days posttreatment, the tumor volume in the control group monotonically increased, while the tumor volumes in the CDDP-only group and PMW-only group did not change greatly and that in the PMW+CDDP group slightly decreased. Afterward, the tumors started to regrow in all treatment groups, but the tumor growth rate was considerably low in the PMW+CDDP group. There was a significant difference in the time courses of tumor volume between the PMW+CDDP group and the control group for up to 14 days posttreatment. The ratio of the Ki-67-positive (proliferative) areas to the whole tumor regions in the PMW+CDDP group was significantly smaller than that in the control group at 7 days posttreatment. These results are attributable to the synergistic effects of enhanced extravasation of CDDP and mechanical tumoricidal effect by PMWs.

**Conclusion:** The combined application of CDDP and PMWs significantly improved the antitumor effects on mouse subcutaneous tumors.

## Introduction

Chemotherapy is the gold standard for cancer treatments, especially when surgery and/or radiation therapy cannot be applied or when the cancer recurs after these treatments. However, the treatment outcome of chemotherapy is not always satisfactory due to the limited efficiency of drug delivery from the bloodstream to tumors. Nonselective delivery of an antitumor agent can also cause serious side effects 1,2. Thus, a drug delivery system (DDS) that enables highly efficient, site-selective delivery of an antitumor agent has recently received much attention 3-7.

Methods using physical energy such as electric pulses (electroporation) and ultrasound (sonoporation) are widely used for site-selective drug delivery in vivo 5-7. For electroporation, however, electrodes need to be inserted into the target tissue, a procedure that is invasive. In addition, the region of discharge is determined by the distribution of electrical impedance in the tissue, which is difficult to control. Sonoporation often requires injection of an enhancer (micro- or nanobubbles), of which the distribution determines the region for drug delivery. The distribution of an enhancer is also difficult to control. In addition, ultrasound should be applied for a certain period of time (e.g., a few tens of seconds to several minutes), causing thermal side effects in the tissue. Overall, site-selectivity for DDS is affected not only by the zone of the physical energy application but also by the uncontrollable factors both in electroporation and sonoporation.

We previously reported site-selective transvascular drug delivery by the use of nanosecond pulsed laser-induced photomechanical waves (PMWs) or laser-induced stress/shock waves; PMWs can enhance the permeability of blood vessels 8. By applying PMW(s) to the skin, muscle and brain in healthy rats, an intravenously administered test drug (Evans blue, EB) could be selectively and efficiently delivered to those targeted tissues 8. This method does not require any auxiliary means, such as application of an enhancer. In addition, the duration of a PMW is very short (less than one microsecond), and only a limited number of pulses (e.g., one to ten pulses) is needed for drug delivery, thus causing no thermal side effect. On the basis of these results, we showed that the method was useful for selective delivery of the test drug to mice with subcutaneous inoculation of human head and neck cancer cells 9. However, the method has not been applied to the delivery of an actual antitumor agent, and the efficacy of the method for tumor treatment is unknown. In this study, we applied PMWs to the delivery of an actual antitumor agent, cisplatin (cis-diamminedichloroplatinum, CDDP), in the same mouse tumor model and examined how useful the method was for enhancing the antitumor effects. CDDP has been used clinically for many years and is the gold standard for chemotherapy of various types of cancer, including head and neck cancer. However, it is difficult to selectively deliver CDDP to tumors by intravascular administration and systemic circulation, and serious side effects often occur 1,2.

## Materials and Methods

### Generation of PMWs

**Figure 1:**
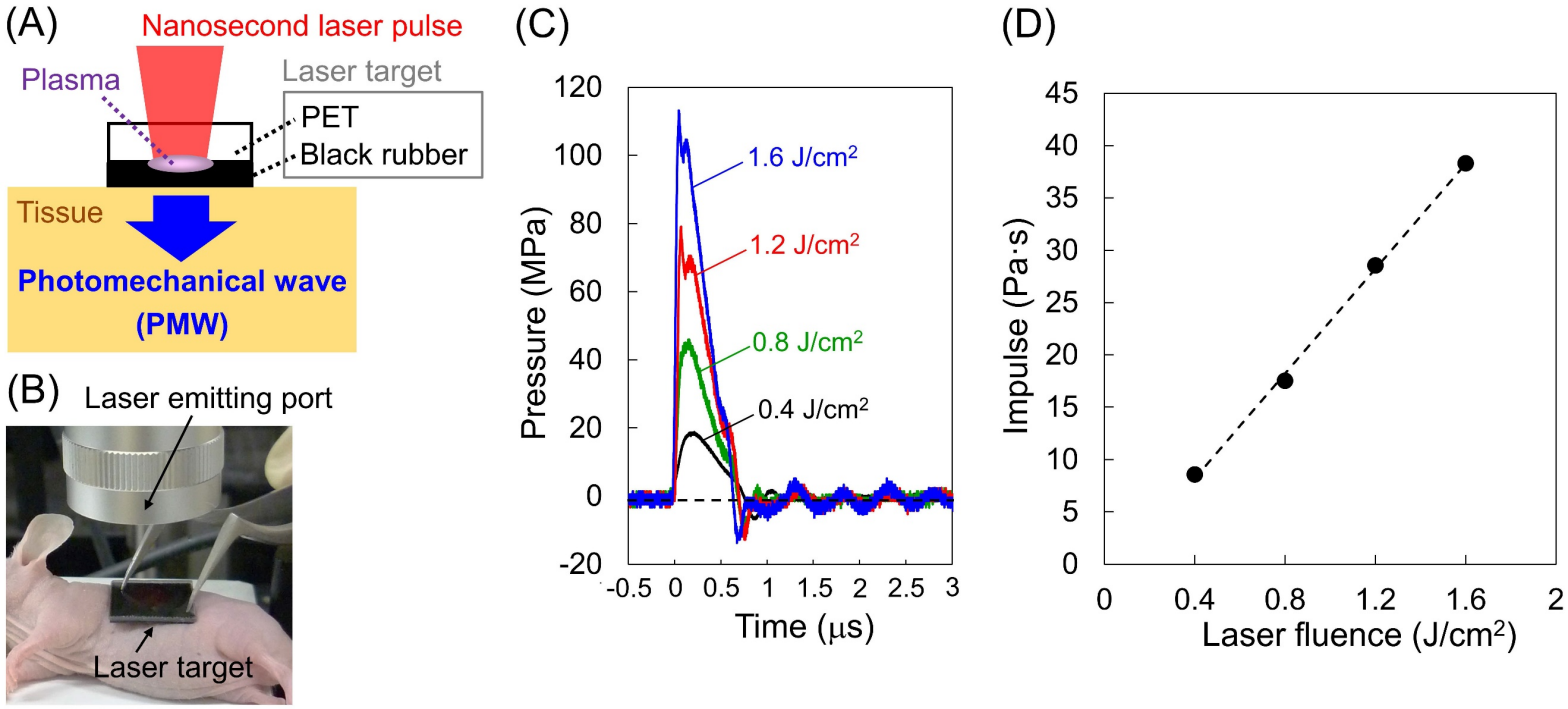
Generation, application and characteristics of PMWs. (A) Schematic diagram for the generation of PMWs. PET, polyethylene terephthalate. (B) Photograph of the setup for PMW application to a subcutaneous tumor in a mouse. (C) Temporal waveforms of PMWs generated at laser fluences of 0.4, 0.8, 1.2 and 1.6 J/cm^2^ with a spot diameter of 6 mm. (D) Correlation between laser fluence and impulse of generated PMWs.

Figure 1 (A) and (B) show a schematic diagram and a photograph of the setup for the generation and application of PMWs to a mouse. A laser target was placed on the skin over the tumor and irradiated with a Q-switched ruby laser pulse (wavelength, 694 nm; pulse width, 20 ns; spot diameter, 6 mm; RH356, JMEC). The laser target consisted of a laser-absorbing material (15-mm-square, 0.5-mm-thick black natural rubber sheet) bonded with an optically transparent material (15-mm-square, 1.0-mm-thick polyethylene terephthalate (PET) sheet) for confining the laser-induced plasma, which can greatly increase the impulse (time-integral positive pressure) of the PMWs generated. Temporal waveforms of PMWs generated at various laser fluences (0.4, 0.8, 1.2 and 1.6 J/cm^2^) were measured with a needle-type hydrophone (frequency band, 0.2-10 MHz; element diameter, 1.0 mm; HNR-1000, ONDA Corp.).

### Animal model

Human head and neck cancer cells (FaDu cells) were used as a standard, stable cancer cell line. The methods for the preparation of the mouse subcutaneous tumor model were described in detail in our previous report 9,10. FaDu cells (5 x 10^6^ cells) were subcutaneously inoculated into the left rear flanks of nude mice (female; 5-7 weeks old; Japan SLC, Inc.) under isoflurane anesthesia, inducing solid tumors.

### Treatments

At seven days after inoculation of FaDu cells, the tumor volume reached ~100 mm^3^. On that day, the mice were divided into four groups: control without any treatment (control), CDDP application only (CDDP only), PMW application only (PMW only) and combined application of PMWs and CDDP (PMW+CDDP). PMWs and/or CDDP were/was applied only at this timepoint (Day0).

A laser target was placed on the skin over the tumor in each mouse, which was under anesthesia by isoflurane inhalation (Figure 1 (B)); between the laser target and the tissue, ultrasound jelly (GEL-SCAN-KA, Hitachi Aloka Medical, Ltd.) was used for acoustic impedance matching. PMWs were generated in the same manner as that used for the pressure measurements described above. We optimized the laser fluence and the number of pulses in terms of the efficiency and safety of the present DDS. The laser target was replaced with a new target for each laser pulse irradiation.

As an antitumor agent, we used CDDP (300.05 Da; Pfizer Inc.), which is a platinum-based antineoplastic drug approved for the treatment of a wide variety of cancers, including head and neck cancer, in clinical practice. A CDDP solution was intraperitoneally injected into the mice at a dose of 2.5 mg/kg. For the PMW+CDDP group, CDDP was administered immediately after PMW application.

### Measurements of tumor volume and body weight

For evaluation of the efficacy and adverse effects of each treatment, the tumor volume and body weight of the mice were measured before treatment and 2, 4, 7, 10 and 14 days after treatment for all groups (n=8). Longitudinal and transverse diameters of the tumors were measured with a digital caliper, and tumor volumes were calculated by the following formula: volume = 4π/3 x (longitudinal diameter/2) x (transverse diameter/2)^2^.

### Histological assessments

Subcutaneous tumors were excised together with the skins at seven days after each treatment. Immediately after excision, the tissues were frozen in an optimal cutting temperature compound (Sakura Finetek, Inc.). After sectioning them into 10‐μm‐thick slices, staining with hematoxylin and eosin (H&E) and nicotinamide adenine dinucleotide‐diaphorase (NADH‐D) and Ki-67 immunofluorescence staining were performed to assess the tissue viability and proliferation capacity of the tumors. With NADH‐D staining, mitochondria in viable cells were stained blue due to their enzyme activity, while those in non-viable cells were not stained 11,12. Tissue sections were incubated in a solution of reduced *b*-NADH (0.8 mg/mL; N8129, Merck KGaA), nitro blue tetrazolium (0.5 mg/mL; N5514, Merck KGaA) and tris-buffered saline (0.05 M, pH 7.6; S3001, Agilent Technologies Inc.) for 30 min at 37℃. Thereafter, the sections were fixed in 10% neutral buffered formalin (062-01661, Fujifilm Wako Pure Chemical Co.) for 30 min. For Ki-67 immunofluorescence staining, after blocking endogenous peroxidase with 0.3% hydrogen peroxide/40% methanol, the tissue sections were incubated with anti-Ki-67 antibody (1:200; RM-9106-S0, Thermo Fisher Scientific Inc.) overnight at 4℃. After washing the sections with phosphate-buffered saline (PBS), they were incubated with DyLight 488 secondary antibody (DI-1488, Vector Laboratories Inc.) for 30 min at room temperature. After washing with PBS, cell nuclei were stained with 4′,6-diamidino-2-phenylindole (DAPI; 0100-20, Southern Biotechnology Associates Inc.). The distributions of viable tumor cells (NADH-D-stained tumor cells) and proliferative tumor cells (Ki-67-positive tumor cells) were observed under a microscope (BZ-X700, Keyence Corp.). As shown in the Results section, the distributions of Ki-67-positive/negative tumor cells vary from region of interest (ROI) to ROI even in the same tissue section. Thus, the ratios of the Ki-67-positive areas to the whole areas of the tumors were evaluated and statistically compared between the groups (n=8).

### Statistical analyses

Statistical analyses were performed with analysis of variance followed by Dunnett's multiple comparison test. *P* values of less than 0.05 were considered statistically significant.

## Results

### Optimum PMW conditions

Figure 1 (C) shows temporal waveforms of PMWs generated at 0.4, 0.8, 1.2 and 1.6 J/cm^2^ with a spot diameter of 6 mm. The impulses (time-integral positive pressures) were 8.6, 17.5, 28.5, and 38.3 Pa·s at 0.4, 0.8, 1.2, and 1.6 J/cm^2^, respectively; the impulse almost linearly increased with increasing laser fluence (Figure 1 (D)). The efficiency of PMW-based transvascular drug delivery is primarily determined by the impulse of the applied PMWs as well as the number of pulses 8. On the other hand, an excessive increase in the PMW impulse can cause tissue damage, such as hemorrhage 8. When the PMWs generated at 1.6 J/cm^2^ were applied to the skins over the subcutaneous tumors in mice, neither apparent injury nor hemorrhage was observed with up to at least 10 pulses. Thus, we chose this condition for the application of PMWs to the animals.

### Tumor volume and body weight

**Figure 2:**
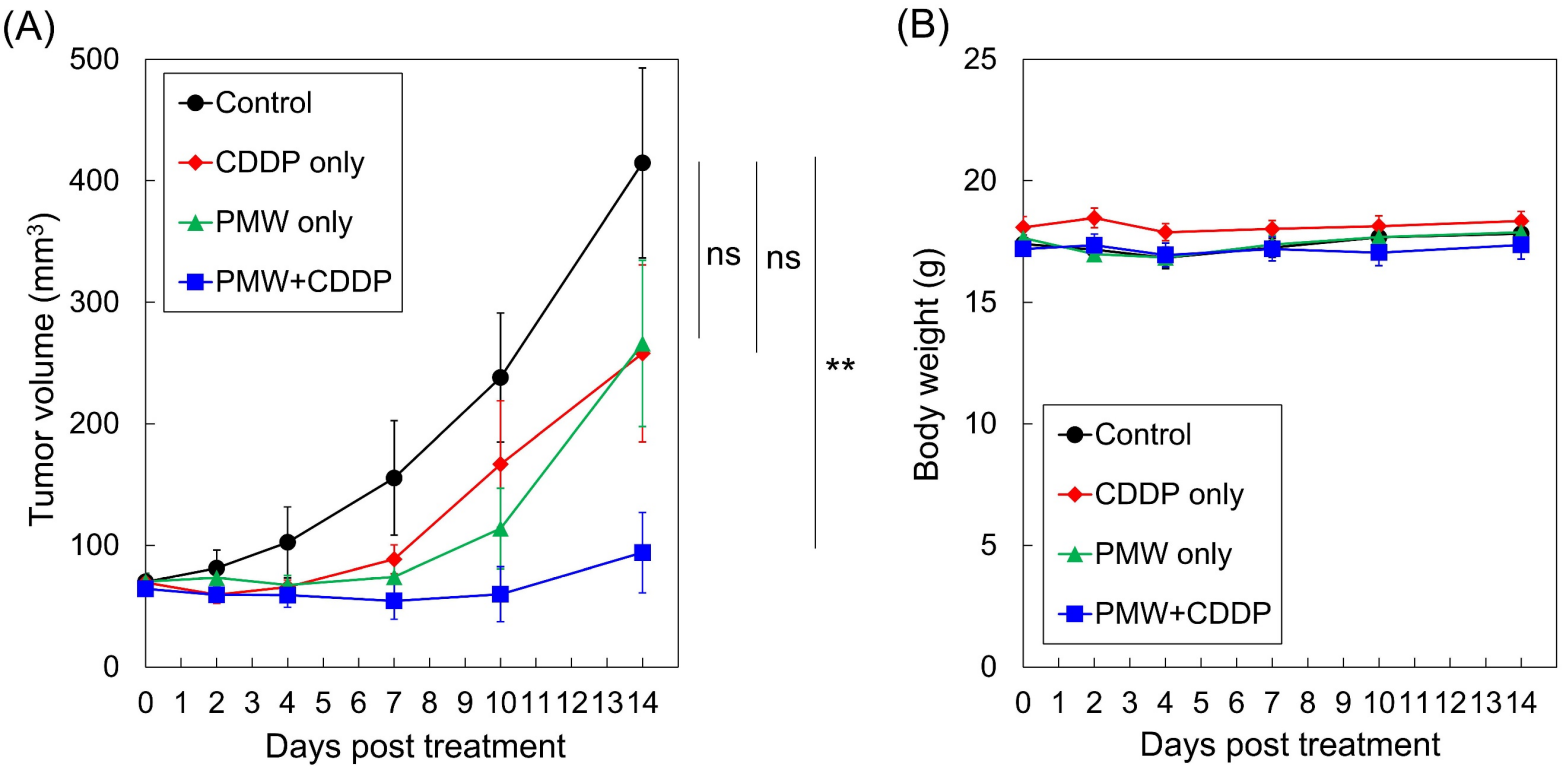
Time courses of (A) average tumor volumes and (B) average body weights in all groups (n=8). Values are expressed as the means ± standard errors. ns, not significant; **, p < 0.01.

Figure 2 (A) shows the time courses of average tumor volumes in all groups. While the tumor volume in the control group monotonically increased, the tumor volumes in the CDDP-only group and PMW-only group did not change greatly until seven days posttreatment (Day 7), showing the retarding effects of these treatments on tumor growth. Thereafter, however, the tumors grew remarkably. The average tumor volumes at Day 14 for these two groups were as large as approximately two-thirds of that for the control group. In the PMW+CDDP group, the tumor volumes slightly decreased until Day 7. Thereafter, the tumors started to slowly regrow, and the growth rate was limited. The average tumor volume at Day 14 was smaller than a quarter of that in the control group; there was a significant difference between the time courses of the tumor volumes in the PMW+CDDP group and control group, indicating a significant antitumor effect of the combined application of PMWs and CDDP. Figure 2 (B) shows the time courses of average body weights in all groups. There was no significant difference between the control group and any treatment group.

### Histological assessments

**Figure 3:**
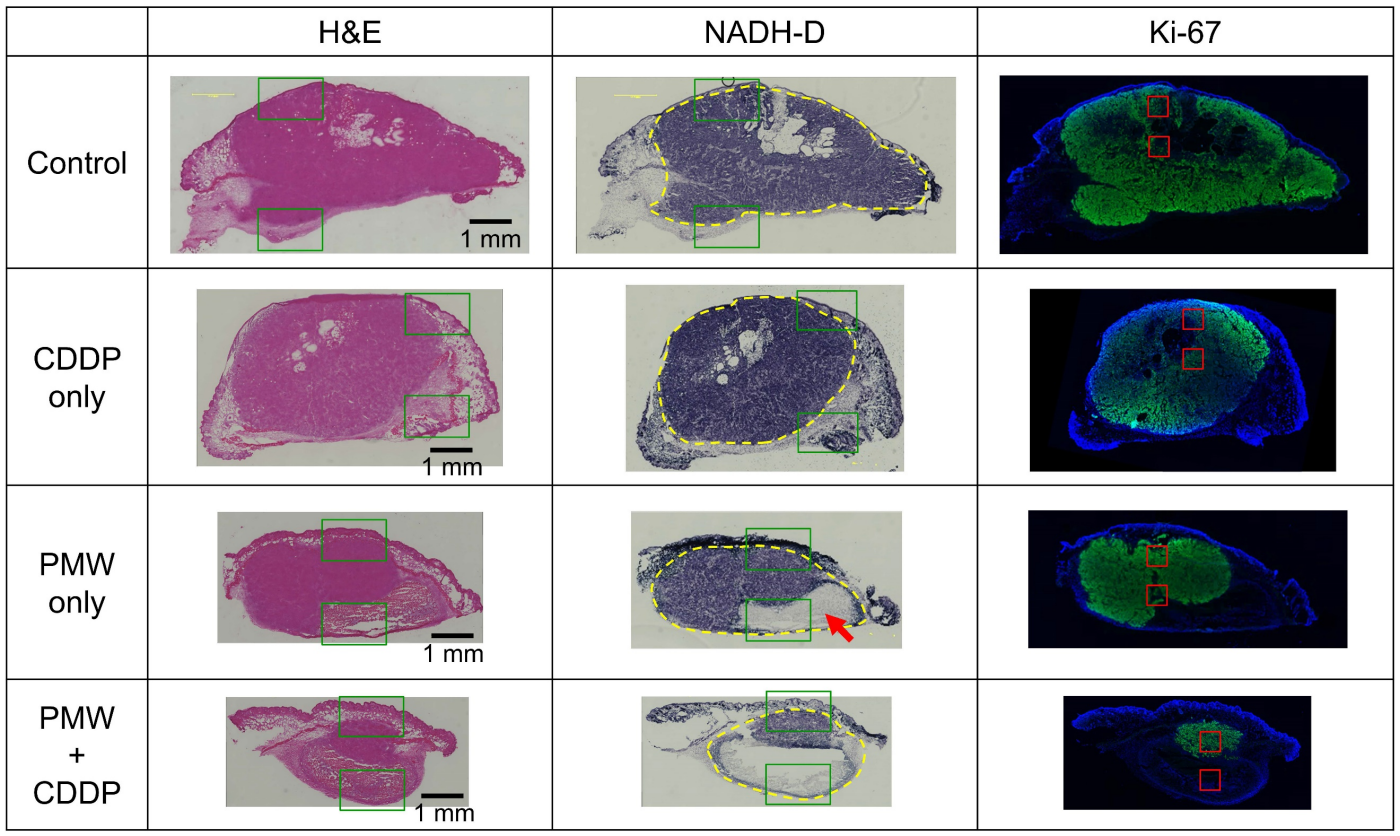
Representative H&E-stained (left), NADH-D-stained (middle) and Ki-67 immunofluorescence-stained (right) cross-sectional images of whole excised tumors with skins at Day 7 in all groups. The yellow dashed lines indicate tumor regions. The red arrow indicates a partially non-viable area in the tumor. The green frames indicate ROIs for high-magnification images shown in Figure 4. The red frames indicate ROIs for high-magnification images shown in Figure 5 (A).

**Figure 4:**
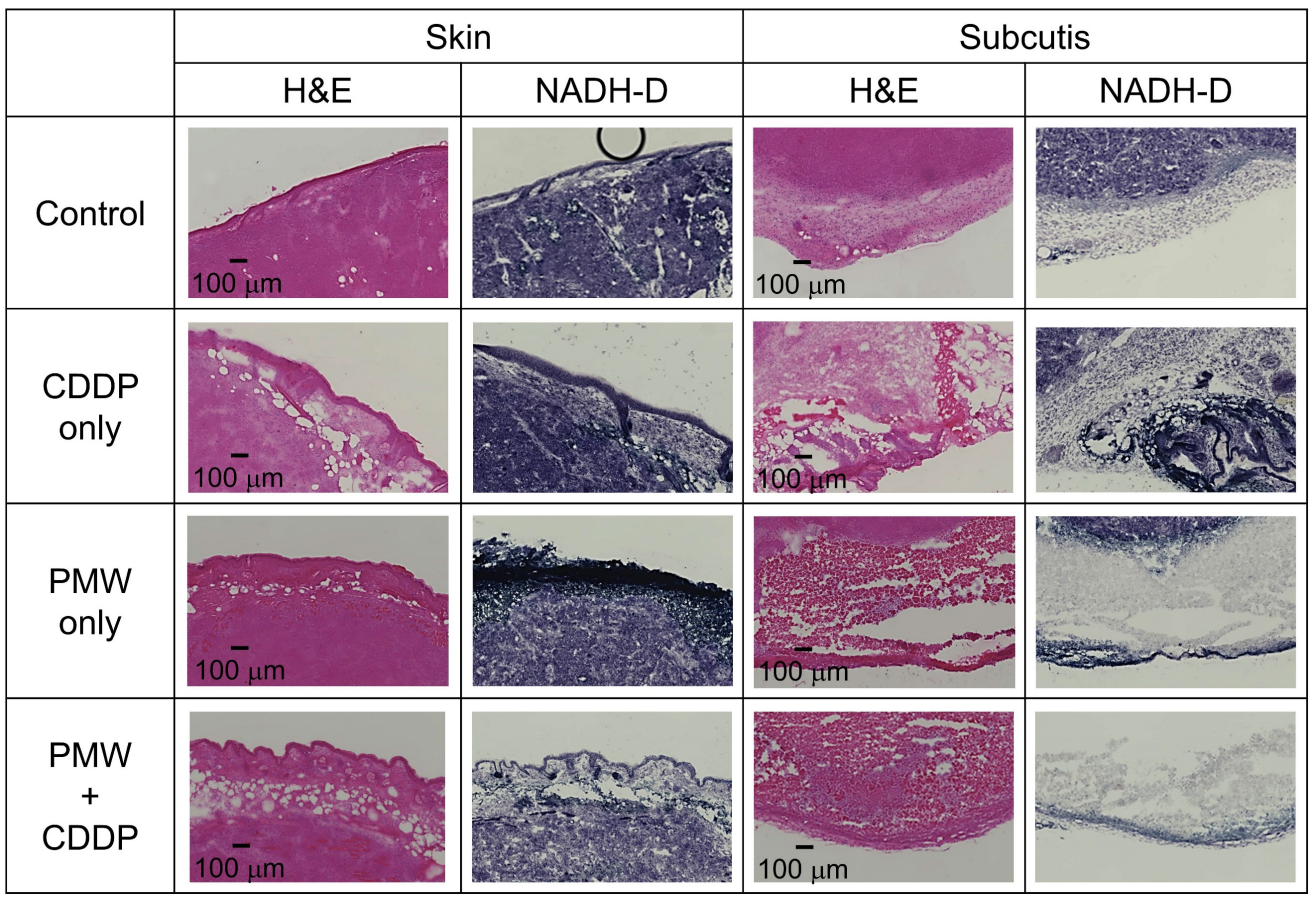
High-magnification images (1.5 x 1.0 mm^2^) of normal skins and subcutis under the tumor stained with H&E and NADH-D in all groups; the ROIs are indicated by green frames in Figure 3.

**Figure 5:**
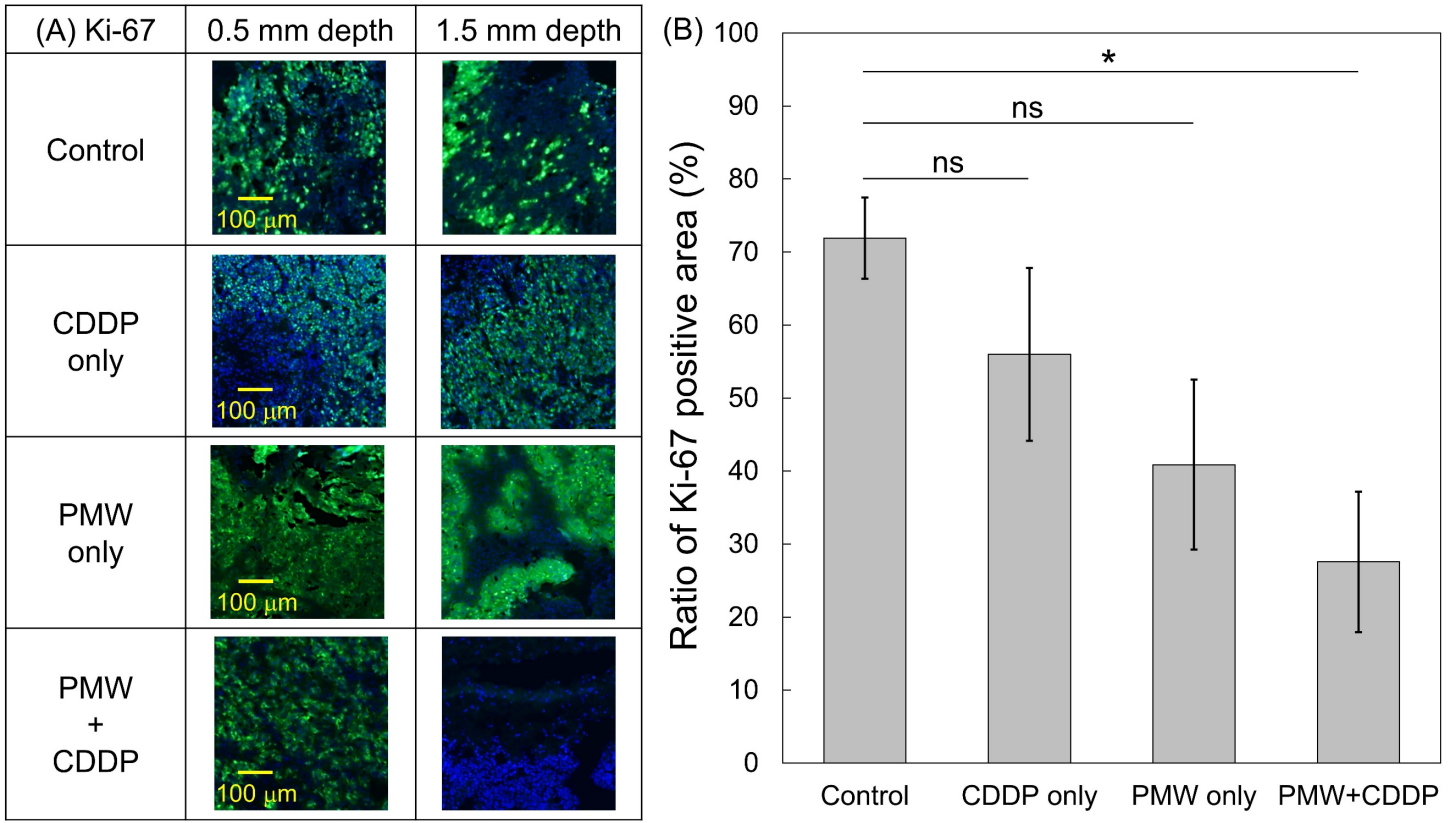
(A) High-magnification images (0.5 x 0.5 mm^2^) for Ki-67 immunofluorescence-stained sections; the ROIs were defined at depths of 0.5 mm and 1.5 mm, respectively, as indicated by red frames in Figure 3. (B) Average ratios of the Ki-67-positive areas to the whole tumor areas in all groups (n=8). Values are expressed as the means ± standard errors. ns, not significant; *, p < 0.05.

Figure 3 shows representative H&E-stained (left), NADH-D-stained (middle) and Ki-67 immunofluorescence-stained (right) cross-sectional images of the whole excised tumors with skins at Day 7; adjacent sections from the same tumor sample were used for each staining. Figure 4 shows high-magnification images (1.5 x 1.0 mm^2^) including normal tissue areas, i.e., skin and subcutis under the tumor, for the H&E-stained and NADH-D-stained sections in Figure 3; the ROIs (1.5 x 1.0 mm^2^) are indicated by the green frames. As shown in the results of tumor volume measurements (Figure 2A), the tumor areas (yellow dashed lines in Figure 3) were greater in the order of the control group, CDDP-only group, PMW-only group and PMW+CDDP group. However, some parts of the tumors were not viable (NADH-D unstained), and the viable/non-viable ratios of the tumors varied depending on the groups. In the control group and the CDDP-only group, most of the tumors were viable. In the PMW-only group, on the other hand, non-viable areas were partially observed in the tumors (red arrow in Figure 3), while no apparent non-viable areas were observed in the skin or non-tumor subcutaneous region (Figure 4). In the PMW+CDDP group, the majority of the tumors were non-viable, indicating massive tumor cell death by the combined treatment. There was no apparent damage in the skin or non-tumor subcutaneous region (Figure 4). According to the Ki-67 immunofluorescence-stained images, the viable (NADH-D stained) tumors were Ki-67 positive, i.e., proliferative, in all groups (Figure 3). Figure 5 (A) shows high-magnification images for Ki-67 immunofluorescence-stained sections in Figure 3; the ROIs (0.5 x 0.5 mm^2^) were defined at depths of 0.5 mm and 1.5 mm, respectively, as indicated by the red frames. Figure 5 (B) shows the average ratios of the Ki-67-positive areas to the whole areas of the tumors in all groups: 71.9% in the control group, 56.0% in the CDDP-only group, 40.9% in the PMW-only group and 27.5% in the PMW+CDDP group. There was a significant difference in the ratios between the PMW+CDDP group and the control group, indicating the efficacy of the combined treatment.

## Discussion

In cancer tissue, the permeability of blood vessels is increased and lymphatic systems are immature, enabling the redistribution of a drug in the bloodstream to the tumor (enhanced permeability and retention [EPR] effect 13). However, the extent of the EPR effect varies depending on the type and location of the tumor, status of blood perfusion and characteristics of a drug 14 and is thus often limited, reducing the efficacy of chemotherapy. Thus, methods for enhancing the transport of a blood-borne drug are eagerly desired. In our previous study using the same model as that used in this study, an intravenously injected test drug (EB) was hardly delivered to the tumor, but application of PMWs to the tumors drastically enhanced the extravasation of EB 9. The molecular weight of EB (960.81 Da) is approximately three-times larger than that of CDDP (300.05 Da), but both drugs efficiently bind to serum albumin (approximately 66,000 Da) in blood 15,16. Thus, there might be a similarity in the pharmacokinetics of the two drugs. In this study, CDDP administration alone led to no significant antitumor effect, while by applying PMWs combined with CDDP administration, the tumor volume was significantly reduced (Figures 2-4). This effect is attributable to the enhanced extravasation of CDDP from the bloodstream to the tumor by PMW application.

We previously evaluated the duration of the transvascular effects of PMW application in the rat normal brain exposed to a PMW using the albumin-EB extravasation method. The results showed that vascular permeability was increased for 8 to 12 hours after the application of a PMW 8. Blood vessels in the normal brain have the blood-brain barrier, while permeability of the tumor vasculature is naturally increased due to the EPR effect. Thus, we expect a longer duration of the increased vascular permeability in the present tumor model than that in the rat normal brain. We plan to experimentally assess the duration of the enhanced vascular permeability in the mouse tumor exposed to PMWs under the current conditions in our next study.

Interestingly, the average tumor volume of the PMW-only group was considerably smaller than that of the control group, although there was no significant difference between them (Figure 2A). This might have been due to the mechanical tumoricidal effect of PMWs; some in vitro studies have shown that a high peak pressure and/or high impulse of PMWs or laser-induced stress/shock waves can induce lethal damage to tumor cells 17,18. To the best of the authors' knowledge, however, there is no report on the antitumor effect of PMWs themselves in vivo. Since PMWs were applied to the tumor through the skin (Figure 1 (B)), the pressure at the skin was highest in the tissue. However, no apparent damage was observed in the skin (Figure 4), indicating higher pressure tolerance of the normal skin tissue than that of the subcutaneous tumor tissue. On the other hand, normal tissue under the tumor should be exposed to pressure that is much lower than that for the tumor because it was shown that PMW pressure was exponentially attenuated in tissue 19. This would be the reason for no apparent damage in the tissue under the tumor (Figure 4). Optimization of PMW conditions, such as the peak pressure and impulse, may lead to a new tumor-selective treatment based on pure photomechanical effects. In this study, the synergistic effects of enhanced extravasation of the drug and mechanical tumor killing by PMWs might have resulted in the significant efficacy of PMW application combined with CDDP administration.

Mice in the PMW-only group showed no significant body weight loss (Figure 2B) with no apparent injury, cell death or hemorrhage in normal tissues as described above, indicating the low invasiveness of the present PMW application. The application of an antitumor drug can cause body weight loss as a side effect. In studies involving CDDP application (5-10 mg/kg, i.p.) to nude mice bearing subcutaneous tumors, drastic body weight losses were observed at ~6 days after drug administration 20,21. In this study, however, mice in the CDDP-only group did not show any decreases in body weights (Figure 2B). This might have been due to the relatively low dose of CDDP (2.5 mg/kg) used. It should be noted that treatment with PMW+CDDP caused significant antitumor efficacy without loss of body weight in the mice. This indicates that PMW application can reduce the necessary dose of an antitumor drug and hence prevent drug side effects.

Although our combined therapy (PMWs + CDDP) showed significant antitumor efficacy, the tumors did not disappear completely, and some proliferative tumor cells survived (Figure 3-5), possibly causing regrowth of the tumors (Figure 2A). Thus, the treatment conditions should be optimized to obtain a complete response. Firstly, there is room for further optimization of PMW conditions such as laser fluence and number of pulses. Secondly, since CDDP is an antitumor agent that can be repeatedly administered in clinical practice and since no side effects of PMW application were shown in this study, repeated application of the present combined therapy could improve the treatment outcome. This will be the most important aspect of the next-step investigation of the present treatment.

Although the pressure of PMWs is attenuated due to absorption and scattering, in addition to the geometrical dissipation in tissue 19, PMWs can be focused in tissue by the use of specially designed geometrical optics, enabling the targeting of deeply located tumors. In our previous study, a PMW was shown to be focused at a depth of ~20 mm in a tissue phantom 22. In addition, PMWs can be generated by the laser transmitted through an optical fiber, enabling endoscopic application of the present method 23,24. As the first clinical targets, we intend to apply this method to squamous cell carcinomas, such as head and neck cancers and esophageal cancers, that develop from the mucosa of the oral cavity, pharynx, larynx and esophagus and can be accessed with a gastrointestinal endoscope. In addition, cancers in other endoscope-accessible organs, such as those in the stomach, colon and rectum, uterine cervix and urinary organs, may be within our scope. However, there are certain limitations in this study. First, the validity of the method was examined for only two weeks posttreatment, and side effects were assessed only on the basis of measurement of body weights. Long-term evaluations of the treatment outcomes, including tumor volume reduction and survival, as well as detailed drug adverse effects, especially for renal function, will be important in the next study. Second, the method was applied only to a single tumor model and to a single antitumor agent. Third, the treatment conditions should be optimized to obtain a complete response, as described above. Considering these issues, we plan to conduct the next study for further validation.

## Abbreviations

CDDP: cis-diamminedichloroplatinum
DAPI: 4',6-diamidino-2-phenylindole
DDS: drug delivery system
EB: Evans blue
EPR: enhanced permeability and retention
NADH-D: nicotinamide adenine dinucleotide‐diaphorase
PBS: phosphate-buffered saline
PET: polyethylene terephthalate
PMW: photomechanical wave
